# Activated clotting time value as an independent predictor of postoperative bleeding and transfusion

**DOI:** 10.1093/icvts/ivae092

**Published:** 2024-05-08

**Authors:** Rafael Maniés Pereira, Diogo Magueijo, Nuno Carvalho Guerra, Catarina Jacinto Correia, Anabela Rodrigues, Ângelo Nobre, Dulce Brito, Luís Ferreira Moita, Tiago R Velho

**Affiliations:** Department of Cardiothoracic Surgery, Hospital de Santa Maria, Centro Hospitalar Lisboa Norte, Lisbon, Portugal; Escola Superior Saúde da Cruz Vermelha Portuguesa, Lisbon, Portugal; Faculdade de Medicina da Universidade de Lisboa, Lisbon, Portugal; Department of Cardiothoracic Surgery, Hospital de Santa Maria, Centro Hospitalar Lisboa Norte, Lisbon, Portugal; Transfusion Medicine Department, Hospital de Santa Maria, Centro Hospitalar Lisboa Norte, Lisbon, Portugal; Transfusion Medicine Department, Hospital de Santa Maria, Centro Hospitalar Lisboa Norte, Lisbon, Portugal; Department of Cardiothoracic Surgery, Hospital de Santa Maria, Centro Hospitalar Lisboa Norte, Lisbon, Portugal; Centro Cardiovascular da Universidade de Lisboa, Faculdade de Medicina, Universidade de Lisboa, Lisbon, Portugal; Centro Cardiovascular da Universidade de Lisboa, Faculdade de Medicina, Universidade de Lisboa, Lisbon, Portugal; Department of Cardiology, Hospital de Santa Maria, Centro Hospitalar Lisboa Norte, Lisbon, Portugal; Innate Immunity and Inflammation Laboratory, Instituto Gulbenkian de Ciência, Oeiras, Portugal; Department of Cardiothoracic Surgery, Hospital de Santa Maria, Centro Hospitalar Lisboa Norte, Lisbon, Portugal; Innate Immunity and Inflammation Laboratory, Instituto Gulbenkian de Ciência, Oeiras, Portugal; Cardiothoracic Surgery Research Unit, Centro Cardiovascular da Universidade de Lisboa (CCUL@RISE), Faculdade de Medicina da Universidade de Lisboa, Lisbon, Portugal

**Keywords:** Activated clotting time, Bleeding, Cardiac surgery, Cardiopulmonary bypass, Postoperative, Transfusion

## Abstract

**OBJECTIVES:**

Activated clotting time (ACT) is commonly used to monitor anticoagulation during cardiac surgeries. Final ACT values may be essential to predict postoperative bleeding and transfusions, although ideal values remain unknown. Our aim was to evaluate the utility of ACT as a predictor of postoperative bleeding and transfusion use.

**METHODS:**

Retrospective study (722 patients) submitted to surgery between July 2018–October 2021. We compared patients with final ACT < basal ACT and final ACT ≥ basal ACT and final ACT < 140 s with ≥140 s. Continuous variables were analysed with the Wilcoxon rank-sum test; categorical variables using Chi-square or Fisher's exact test. A linear mixed regression model was used to analyse bleeding in patients with final ACT < 140 and ≥140. Independent variables were analysed with binary logistic regression models to investigate their association with bleeding and transfusion.

**RESULTS:**

Patients with final ACT ≥ 140 s presented higher postoperative bleeding than final ACT < 140 s at 12 h (*P* = 0.006) and 24 h (**P = 0.004). Cardiopulmonary bypass (CPB) time [odds ratio (OR) 1.009, 1.002–1.015, 95% confidence interval (CI)] and masculine sex (OR 2.842,1.721–4.821, 95% CI) were significant predictors of bleeding. Patients with final ACT ≥ 140 s had higher risk of UT (OR 1.81, 1.13–2.89, 95% CI; *P* = 0.0104), compared to final ACT < 140 s. CPB time (OR 1.019,1.012–1.026, 95% CI) and final ACT (OR 1.021,1.010–1.032, 95% CI) were significant predictors of transfusion. Female sex was a predictor of use of transfusion, with a probability for use of 27.23% (21.84–33.39%, 95% CI) in elective surgeries, and 60.38% (37.65–79.36%, 95% CI) in urgent surgeries, higher than in males.

**CONCLUSIONS:**

Final ACT has a good predictive value for the use of transfusion. Final ACT ≥ 140 s correlates with higher risk of transfusion and increased bleeding. The risk of bleeding and transfusion is higher with longer periods of CPB. Males have a higher risk of bleeding, but females have a higher risk of transfusion.

## Graphical Abstract

**Figure:**
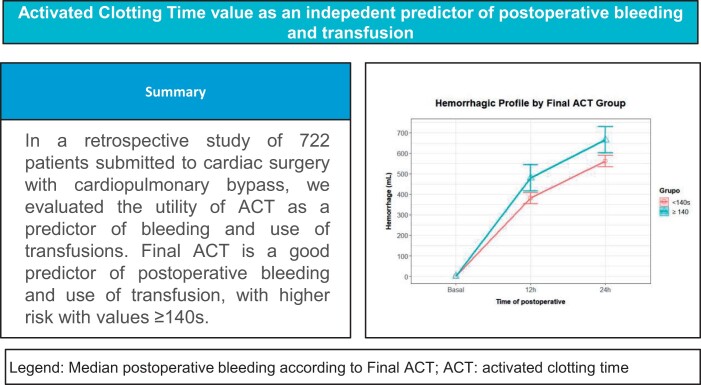

## INTRODUCTION

Cardiac surgery carries a substantial risk of perioperative bleeding, due to the invasiveness of the procedure, the necessity for high-dose anticoagulation, the use of hypothermia and cardiopulmonary bypass (CPB) and the prevalent use of preoperative anticoagulants and antiplatelets medications, among other factors [1]. Up to 60% of the patients submitted to cardiac surgery require allogenic blood transfusions, with cardiovascular surgeries being the top indications for the use of transfusions worldwide [2–4]. The use of transfusions is associated with a tremendous economic burden and a significant increase in morbidity, as well as short and long-term mortality [1, 5, 6]. Consequently, strategies aimed at reducing transfusion use have the potential to improve patient outcomes and decrease costs [1].

Tight control in haemostasis reduces the use of transfusions and related complications, enhancing patient outcomes and mitigating healthcare-associated costs [1]. To achieve this, individualized therapies using point-of-care tests, such as the activated clotting time (ACT), are recommended, as they have been associated with improved patient outcomes compared to standard laboratory-based tests [5].

ACT is the most used test in cardiac surgery to monitor the degree of anticoagulation [7, 8] since it (i) only needs a small sample; (ii) results are promptly available; (iii) minimizes errors associated with samples mislabelling or mishandling; (iv) uses whole blood; and (v) does not need to be performed by a specialized technician [7].

ACT is measured in seconds, and normal values in the absence of heparin usually range from 70 to 150 s, depending on the device used [9]. For surgeries with CPB, the standard recommendation is to achieve ACT values higher than 480 s [10, 11]. ACT is also employed to monitor heparin’s reversal effect. However, despite some suggestions in the literature that a final post-protamine ACT value lower than the pre-heparin value might be advantageous in reducing bleeding and transfusion use [12], few data suggest a concrete cut-off value for the ideal ACT value after protamine administration [1, 8, 10], and its management is currently approached empirically. Postoperative ACT values may be a valuable tool to predict and manage postoperative bleeding, with their optimization being crucial for reducing transfusion use.

The primary objective was to characterize the association between ACT after protamine administration and the need for transfusion, as well as to investigate whether there is a specific ACT value linked a lower risk of bleeding and/or the use of transfusions.

## METHODS

### Patients

The study was approved by the Ethics Committee of Centro Académico de Medicina de Lisboa (Reference No 386/21 on 17 March 2022). Data were retrospectively collected from our Cardiothoracic Surgery Department database. We have included patients between January 2018 and October 2021 with at least 18 years old and submitted to cardiac surgery requiring CPB. Patients under anticoagulation and/or anti-platelet therapies, with postoperative haemorrhagic complication (e.g. surgical re-exploration); and without complete postoperative clinical records have been excluded (Supplementary Material, Fig. S1). Due to the retrospective and anonymous nature of the study, informed signed consent from patients was waived. The study followed the Strengthening the Reporting of Observational Studies in Epidemiology guidelines.

### Surgical technique

For CPB initiation, patients were anticoagulated with a 300 IU/kg dose of sodium heparin, to obtain a target ACT >480 s. CPB with non-pulsatile flow was used in all patients, with a flow indexed to the patient's body surface of 2.4 l/min/m^2^.

Surgeries were performed with mild hypothermia or normothermia (32–36°C, measured in the oropharynx). The allowed minimum haematocrit value during CPB was 24%. After completion of CPB, heparin was reversed with protamine sulphate at a 1:1 ratio, considering the upper limit of 140 s as an adequate postoperative ACT value [12]. We have considered: (i) basal ACT as the ACT value before heparin administration and (ii) final ACT as the value obtained after protamine administration. ACT values were assessed resorting to Medtronic’s HMS Plus Hemostasis Management System (Minneapolis, USA), using High Range ACT (HR-ACT) kaolin 12% cartridges. Drainage of mediastinal blood in the postoperative period was performed using at least 2 drains, one in the anterior mediastinum and the other in the pericardium, with 28 French silicone drainage tubes. The chest tubes were both connected to a drainage bottle with saline solution, connected to a vacuum system for continuous siphon drainage. All patients were transferred to the intensive care unit after surgery and remained with mediastinal drains for, at least, 48 h. Patients were then categorized according to the severity of postoperative bleeding using the criteria defined by Dyke *et al.* [13], considered significant with at least 600 ml in the first 12 h. The use of blood or blood products transfusion was decided according to the criteria described in the European Guidelines on patient blood management for adult cardiac surgery [1].

### Statistical analysis

In the case of missing values relating to the different dependent quantitative variables, the absent values were replaced by the median of the respective variable. Since the number of transfusions does not follow a normal distribution, they were categorized according to their need (needed/not needed). No sample size or statistical power calculations were performed for this work. Continuous variables were expressed as median and interquartile range and analysed using the Wilcoxon rank-sum test, with continuity correction. Categorical variables were presented in the form of absolute frequency and percentages, analysed using the Chi-square test with Yates' continuity correction or Fisher's exact test. All tests were bilateral. A linear mixed regression model with fixed and random effects, adjusted by the restricted maximum likelihood method, was applied to analyse bleeding after surgery in 2 groups: final ACT < 140 s and final ACT ≥ 140 s. The dependent variable was bleeding volume, and the independent variables were ‘group’ (final ACT <140 s or final ACT ≥140 s) and ‘time’ (baseline, 12 h postoperatory, and 24 h postoperatory), as well as the interaction between the latter 2. All model assumptions were verified, namely the absence of multicollinearity of all variables involved in the model and the normality of the model's residuals. The random effects, in this case, include individual variations in the intercepts of the individuals in the sample (identified by ID), allowing the model to account for heterogeneity among individuals.

In addition, 2 binary logistic regression models were used to analyse the association between multiple independent variables (age; sex; body mass index; CPB time; basal ACT; post-heparin ACT; final ACT; difference between final ACT and basal ACT; final ACT relative to 140 s; preoperative fibrinogen; preoperative haematocrit; preoperative platelets; preoperative prothrombine time (PT); preoperative activated partial thromboplastin time (aPTT); hypertension; surgery timing; surgery type and their interactions; diabetes; dyslipidaemia; and smoking) and the dependent variables (significant bleeding and the use of packed red blood cells (PRBC)). To reduce bias, interactions between different types of priming and surgery were included. We started with a simple model and then proceeded to automatically select variables using the Akaike information criterion method.

The specificity and sensitivity of the statistical models used were evaluated resorting to receiver operating characteristic (ROC) curves and the calculation of the area under the curve (AUC).

A *P*-value of <0.05 was considered statistically significant. Statistical analysis was performed using the R Studio software, using the R Base, pROC and nmle packages [14].

## RESULTS

### Patients

Demographic data of 722 included patients is described in Table 1. Almost 55% of the patients were male and the median age was 71 years (interquartile range 63–76). Single non-coronary artery bypass graft (CABG) was the most common surgery (53.4%), followed by 2 procedures (38.5%). Surgery on aorta was performed in 17.6% of the patients.

**Table 1:** Demographic data of the studied population

	All patients	Nonsignificant bleeding	Significant bleeding	*P*-value	No transfusion	Transfusion	*P*-value	Female	Male	*P*-value
*N*	722	631	91		518	204				
Age (years), median (IQR)	71 (63–76)	70 (63–76)	73 (66–78)	0.07	70 (62–75)	73 (65.8–78.3)	**<0.001**	70 (65–69.2)	70 (62–68.1)	0.11
Male, *n* (%)	397 (55)	331 (52.5)	66 (72.5)	**<0.001**	333 (64.3)	64 (31.4)	**<0.001**	—	—	—
Surgery, *n* (%)				**<0.001**			0.081			0.08749
Isolated CABG	19 (2.6)	16 (2.5)	3 (3.3)		14 (2.7)	5 (2.4)		4 (1.2)	15 (3.8)	
Single non CABG	385 (53.4)	353 (56)	32 (34.8)		289 (56)	96 (46.6)		184 (57)	201 (50.4)	
2 procedures	278 (38.5)	235 (37.3)	43 (46.7)		189 (36.6)	89 (43.2)		117 (36.2)	161 (40.4)	
3 procedures	40 (5.5)	26 (4.2)	14 (15.2)		24 (4.7)	16 (7.8)		18 (5.6)	22 (5.4)	
Aorta surgery, *n* (%)	127 (17.6)	106 (16.8)	21 (23.1)	0.19	91 (17.6)	36 (17.6)	1	40 (12.3)	87 (21.9)	**0.001**
Surgical timing, *n* (%)				0.06			**<0.001**			0.482
Elective	688 (95.3)	605 (95.9)	83 (91.2)		503 (97.1)	185 (90.7)		312 (96)	376 (94.7)	
Urgent	34 (4.7)	26 (4.1)	8 (8.8)		15 (2.9)	19 (9.3)		13 (4)	21 (5.3)	
Diabetes, *n* (%)										0.9608
Non-diabetic	526 (72.9)	458 (72.6)	68 (74.7)	0.57	388 (74.9)	138 (67.6)	0.11	238 (73.2)	288 (72.6)	
NIT type II	179 (24.8)	159 (25.2)	20 (22)		120 (23.2)	59 (28.9)		80 (24.6)	99 (24.9)	
IT type II	17 (2.3)	14 (2.2)	3 (3.3)		10 (1.9)	7 (3.5)		7 (2.2)	10 (2.5)	
Dyslipidaemia, *n* (%)	471 (65.2)	409 (64.8)	62 (68.1)	0.25	344 (66.4)	127 (62.3)	0.33	208 (64)	263 (66.2)	0.5808
Hypertension, *n* (%)	589 (81.6)	511 (81)	78 (85.7)	0.35	418 (80.7)	171 (83.8)	0.38	266 (81.8)	323 (81.4)	0.9433
CKD, *n* (%)				**0.026**			**0.005**			0.9965
No (eGFR > 90 ml/min/1.73 m²)	602 (83.4)	532 (84.3)	70 (76.9)		444 (85.7)	158 (77.5)		272 (83.7)	330 (83.1)	
Mild (60–89 ml/min/1.73 m²)	54 (7.5)	42 (6.7)	12 (13.2)		39 (7.5)	15 (7.4)		23 (7.1)	31 (7.8)	
Moderate (30–59 ml/min/1.73 m²)	46 (6.4)	42 (6.7)	4 (4.4)		27 (5.2)	19 (9.3)		21 (6.5)	25 (6.3)	
Severe (15–29 ml/min/1.73 m²)	13 (1.8)	11 (1.7)	2 (2.2)		5 (1)	8 (3.9)		6 (1.8)	7 (1.8)	
Dialysis (eGFR < 15 ml/min/1.73 m²)	7 (0.9)	4 (0.6)	3 (3.3)		3 (0.6)	4 (1.9)		3 (0.9)	4 (1)	
LV ejection fraction, *n* (%)										0.2294
Normal (>50%)	622 (86.2)	551 (87.3)	71 (78)	0.07265	446 (86.1)	176 (86.3)	0.6586	288 (88.6)	334 (84.1)	
Mildly reduced (41–50%)	54 (7.5)	45 (7.1)	9 (9.9)		39 (7.6)	15 (7.4)		19 (5.9)	35 (8.9)	
Moderately reduced (31–40%)	31 (4.3)	23 (3.7)	8 (8.8)		22 (4.2)	9 (4.4)		13 (4)	18 (4.5)	
Reduced (21–30%)	14 (1.9)	11 (1.7)	3 (3.3)		11 (2.1)	3 (1.5)		4 (1.2)	10 (2.5)	
Severely reduced (<21%)	1 (0.1)	1 (0.2)	0 ()		0 ()	1 (0.4)		1 (0.3)	0 (0)	

Statistically significant values are presented in bold. CABG: coronary artery bypass graft; CKD: chronic kidney disease; eGFR: estimated glomerular filtration rate; IQR: interquartile range; IT: insulin treated; NIT: non-insulin treated;

In the subgroup analysis, we observed statistically significant differences between patients with or without significant bleeding in terms of sex (*P* < 0.001), coronary surgery (*P* = 0.01355) and chronic kidney disease (CKD) (*P* = 0.02598). Similarly, we found statistically significant differences between patients with and without the use of transfusion in relation to sex (*P* < 0.001), surgery timing (*P* < 0.001), CKD (*P* = 0.004925) and age (*P* < 0.001).

Table 2 presents perioperative surgical variables and laboratory test results. Although there were no statistically significant differences between patients with or without significant bleeding, noteworthy distinctions emerged when comparing patients with or without the use of transfusion, specifically preoperative haemoglobin, haematocrit, fibrinogen, platelets, basal ACT and final ACT and CPB time (Table 2).

**Table 2:** Median preoperative laboratory values and surgery-dependent variables

	All patients	Non-significant bleeding	Significant bleeding	*P*-value	No transfusion	Transfusion	*P*-value	Male	Female	*P*-value
Haemoglobin (g/dl), median (IQR)	13.5 (12.5–14.6)	13.5 (12.5–14.6)	13.4 (12.35–14.35)	0.1893	13.9 (13–14.8)	12.35 (11.3–13.3)	**<0.001**	14.1 (13.1–14.9)	12.9 (12–13.6)	**<0.001**
Haematocrit (%), median (IQR)	40.2 (37.2–42.8)	40.2 (37.2–42.9)	39.4 (36.85–42.3)	0.1651	41.3 (38.62–43.7)	36.9 (34–39.7)	**<0.001**	41.7 (38.8–44)	38.4 (36.1–40.7)	**<0.001**
Fibrinogen (mg/dl), median (IQR)	333 (280–394.8)	333 (283–395)	330 (260–388.5)	0.2413	333 (276–378.8)	347 (300.5–433.5)	**<0.001**	333 (273– 381)	333 (293–400)	**0.0217**
Platelets (×10^9^/l), median (IQR)	213 (177–255)	213 (177.5–257)	207 (173.5–234)	0.9659	206 (174.2–247.8)	220.5 (189–273.8)	**0.0013**	199 (166 –239)	224 (193– 272)	**<0.001**
PT (s), median (IQR)	12.1 (11.5–12.78)	12.1 (11.5–12.7)	12.1 (11.6–12.95)	0.3419	12.1 (11.6–12.7)	12.1 (11.5–12.9)	0.79	12.1 (11.6–12.9)	12 (11.5–12.6)	0.0193
aPTT (s), median (IQR)	29 (27–31)	29 (27–31.05)	29 (27.25–30.8)	0.9755	29 (27.1–30.98)	29 (26.6–31.3)	0.7509	29 (27.2–31.4)	29 (26.9–30.7)	0.2131
Basal ACT (s), median (IQR)	140 (126–150)	140 (126–150)	140 (130.5–151.5)	0.5786	139 (126–149)	142 (126–154)	**0.0381**	140 (129–151)	139 (123–150)	0.0516
Post-heparin ACT (s), median (IQR)	488 (428–558)	488 (425–558)	498 (452.5–575.5)	0.1667	488 (424–553)	493.5 (433–563.2)	0.3373	493 (428–568)	483 (428–549)	0.2229
Post-protamine ACT (s), median (IQR)	121 (110.2–132)	121 (110–132)	123 (112–135)	0.4023	120.5 (109–131)	125.5 (114.8–138)	**<0.001**	121 (109–131)	122 (112–134)	0.0555
CPB time (min), median (IQR)	62 (109–131)	62 (43–78)	74 (50.5–92.5)	0.00165	61 (42.25–79)	66 (49–84)	**0.0190**	68 (46–86)	59 (41–75)	**<0.001**
Ao-cross time (min), median (IQR)	49 (33–64)	48 (32–62.5)	56 (39–75.5)	**<0.001**	48.5 (32–63)	49 (37–66)	0.0846	53 (36–66)	45 (30–57)	**<0.001**

Statistically significant values are presented in bold. ACT: activated clotting time; Ao-cross: aortic cross-clamp; aPTT: activated partial thromboplastin time; CPB: cardiopulmonary bypass; IQR: interquartile range; PT: prothrombine time.

### Activated clotting time and haemorrhage

**Figure 1:**
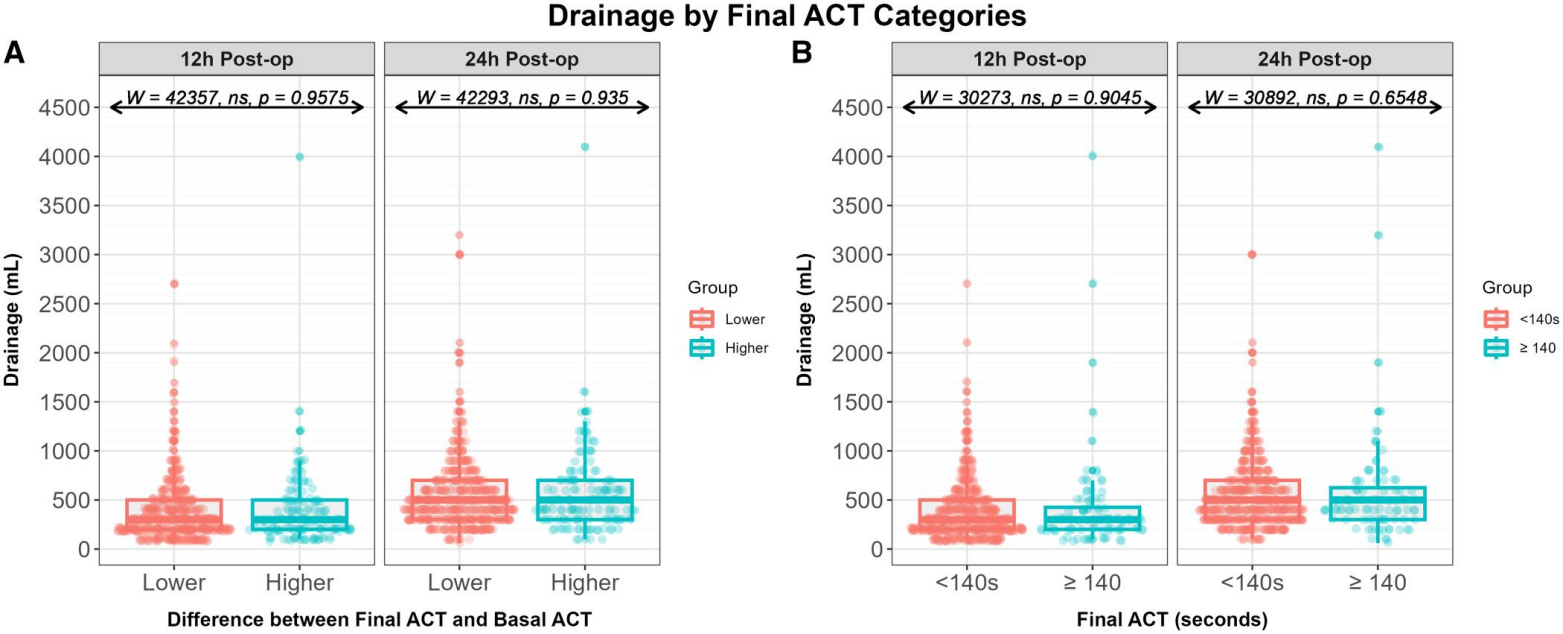
Postoperative bleeding at 12 and 24 h according to (**A**) differences between final and basal ACT and (**B**) final ACT <140 or ≥140 s. ACT: activated clotting time.

To investigate the relationship between ACT and Haemorrhage, we started by assessing blood drainage at 12 and 24 h postoperatively among patients with higher or lower final ACT compared to their basal value (Fig. 1). No statistically significant differences were observed at 12 h (*W* = 42 357, ns, *P* = 0.9575) or 24 h (*W* = 42 293, ns, *P* = 0.935) (Fig. 1). Afterwards, using the reference value of 140 s, we compared postoperative bleeding at 12 and 24 h among patients with a final ACT lower or higher than 140 s. No statistically significant differences were found at 12 h (*W* = 30 273, ns, *P* = 0.9045) and 24 h (*W* = 30 892, ns, *P* = 0.6548) (Supplementary Material, Fig. S2). Moreover, no statistically significant differences were observed among patients with significant bleeding and final ACT < 140 s (10.8%) or with ≥140 s (1.8%) (χ^2^ = 0.0174, df = 1, *P* = 0.8948).

We then explored the relationship between final ACT and postoperative bleeding (Supplementary Material, Fig. S2). Time has a strong positive effect, with the effect at 12 h of 379 384 ± 14 187 (***P < 0.001) and 560 088 ± 14 188 (***P < 0.001) at 24 h (Supplementary Material, Table S1).

**Figure 2:**
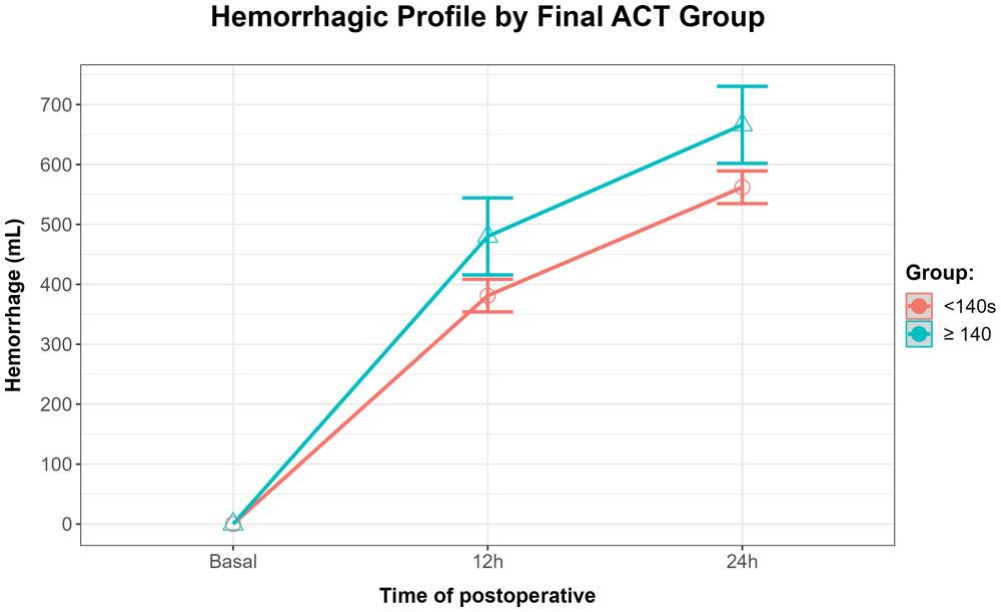
Median postoperative bleeding of patients with final ACT ≥140 s compared to <140 s at 12 and 24 h after surgery. ACT: activated clotting time.

According to the model, the estimated mean bleeding at 12 h postoperatively for a patient with final ACT < 140 s is 379.38 ± 14.19 ml, increasing to 560.09 ± 14.19 ml 24 h after surgery. For a patient with final ACT ≥ 140 s, the estimated postoperative bleeding was 479 ± 36.39 ml at 12 h and 665.09 ± 36.39 ml at 24 h after surgery (Supplementary Material, Table S2). On average, a patient with a final ACT ≥ 140 s had a statistically significant increase of postoperative bleeding of 99.62 ± 36.39 ml in the first 12 h, and 104.99 ± 36.39 ml 24 h after surgery, compared to a patient with final ACT < 140 s. Moreover, patients with final ACT ≥ 140 s exhibited statistically significant higher bleeding levels at 12 and 24 h, despite the fact that the bleeding rate was higher in the first 12 h, and similar in both groups in the following 12 h (between 12 and 24 h postoperatory) (Fig. 2).

To further explore our results, we evaluated the relationship between significant bleeding (dependent variable), and several other possibly explanatory variables. The final model included the following explanatory variables: (i) CPB time (χ^2^ = 7.6850, df = 1, **P = 0.005568), with a positive coefficient, associated with an increased risk of significant bleeding; (ii) preoperative haematocrit (χ^2^ = 8.7867, df = 1, **P = 0.003034), with a negative coefficient, indicating that an increase is associated with a decrease in postoperative bleeding; and (iii) patient sex (χ^2^ = 15.9175, df = 1, ***P < 0.001), with a positive coefficient for male, indicating a higher bleeding risk compared to females (Supplementary Material, Table S3). All other variables, including final ACT and others describing ACT changes, were not significant in the final model.

**Figure 3:**
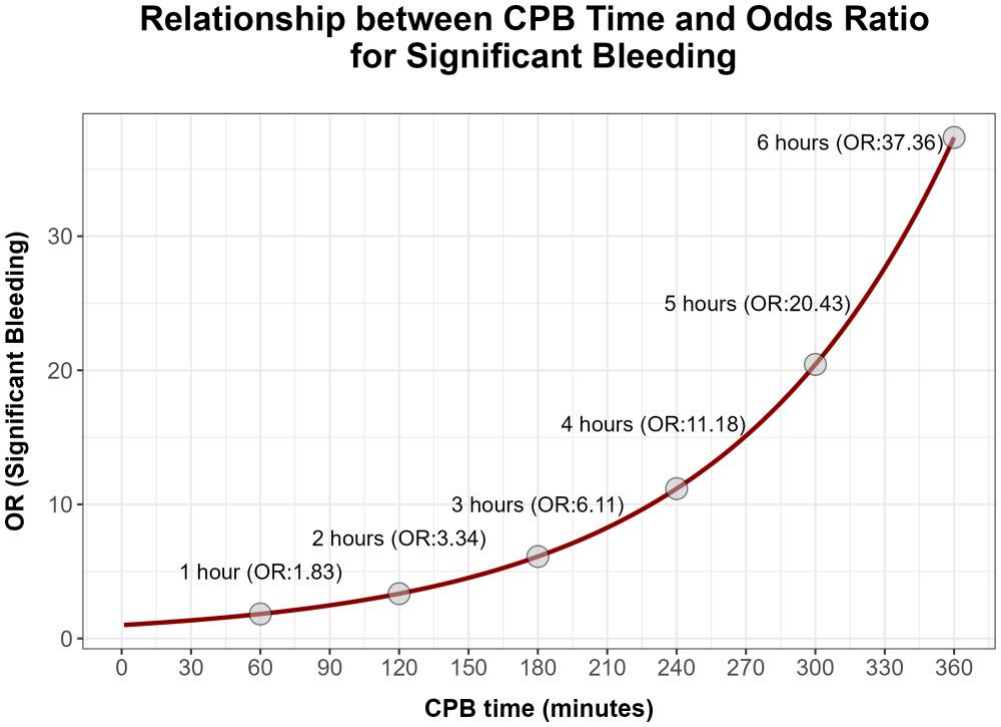
Visual representation of the relation between odds ratio (OR) for significant bleeding and the duration of cardiopulmonary bypass.

The odds ratio (OR) for the variable ‘CPB time’ is 1.009 [1.002–1.015, 95% confidence interval (CI)], representing that with each minute of CPB, the OR for the presence of significant bleeding increases 1.009 times, assuming that the other variables are constant. Figure 3 illustrates the exponential increase in OR for the CPB time, while maintaining the remaining variables constant. For a CPB time of 1 h, the OR is 1.83, but almost doubles with another hour of CPB (2 h, OR 3.34), and triples with 3 h (OR 6.11), increasing up to 37.36 with 6 h of CPB.

**Figure 4:**
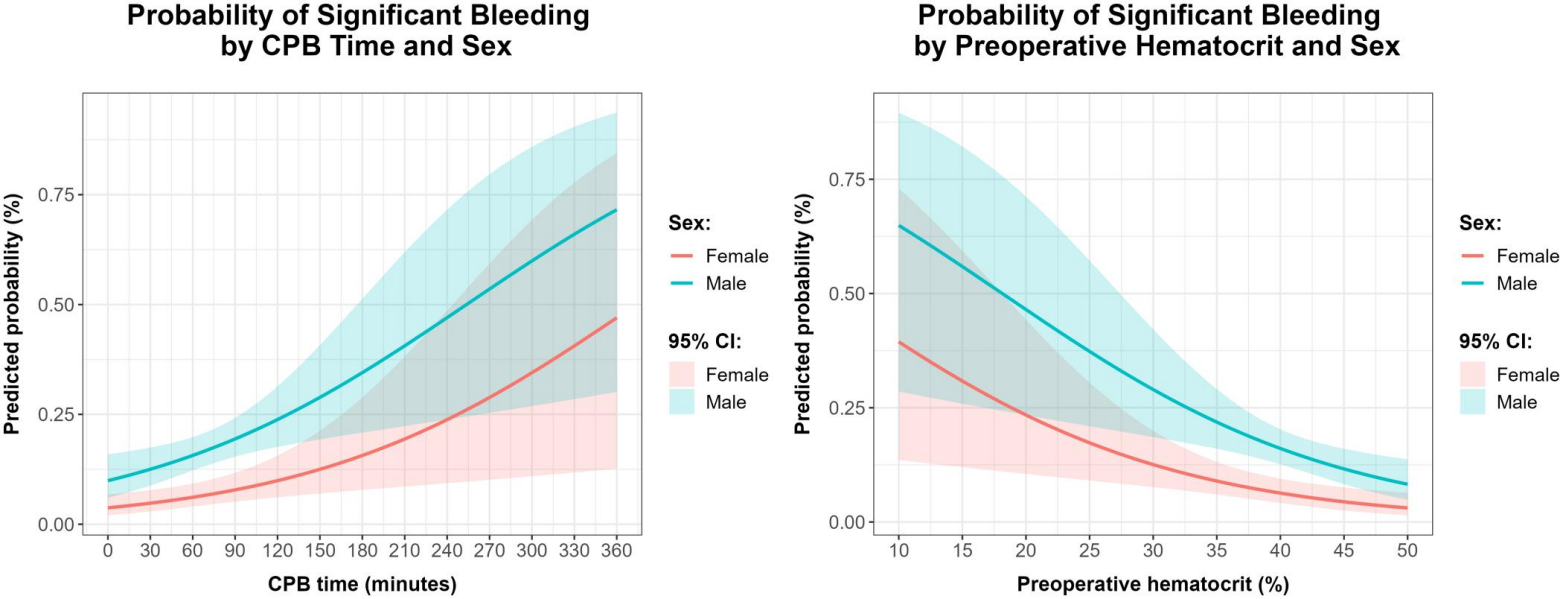
Line chart with 95% confidence interval bands with the relation between significant bleeding probability and (**A**) cardiopulmonary time for males (blue) and females (pink) or (**B**) preoperative haematocrit for males (blue) and females (pink).

Predicted probabilities for the model’s dependent variable (significant bleeding) by patient sex were also estimated for patients with a CPB time and preoperative haematocrit that correspond to their respective median value (62 min and 40.2%, respectively). For a female patient, the predicted probability of significant bleeding is, according to this model, 6.23% (4.10–9.36%, 95% CI), while a male with the same variables has a predicted probability of significant bleeding of 15.89% (12.47–20.02%, 95% CI). Figure 4 represents the predicted probability of significant bleeding by CPB time and patient sex. Males are at a higher risk of significant bleeding after cardiac surgery, on average 2.842 (1.721–4.821, 95% CI) times higher. The same is valid with a preoperative haematocrit between ∼32% and 46%, with a significantly higher predicted postoperative bleeding in males (Fig. 4), assuming the median CPB time. Furthermore, while statistically different, the probability of significant bleeding in men and women is lower for higher values of haematocrit.

A ROC curve was elaborated for the binary logistic regression model (Supplementary Material, Fig. S3), yielding an AUC value of 0.702.

### Activated clotting time and use of transfusion

We subsequently investigated the association between the use of at least 1 PRBC and final ACT, dividing patients into 2 groups (<140 or ≥140 s). A final ACT ≥ 140 s is associated with a higher need for PRBC transfusion (*P* = 0.0104), with an estimated OR of 1.81 (1.13–2.89, 95% CI).

We used the approach previously described to further explore variables associated with the use of at least 1 PRBC. The final model included the following explanatory variables: CPB time (χ^2^ = 29.5481, df = 1, ***P < 0.001), final ACT (χ^2^ = 12.8903, df = 1, ***P ≤ 0.001), preoperative haematocrit (χ^2^ = 93.0216, df = 1, ***P < 0.001), surgery timing (χ^2^ = 8.5668, df = 1, **P = 0.0034), age (χ^2^ = 6.6213, df = 1, *P = 0.01) and patient sex (χ^2^ = 24.5074, df = 1, ***P < 0.001). Quantitative explanatory variables CPB time, final ACT, surgery timing (urgent surgery) and patient age generated positive coefficients, indicating a higher risk of using at least 1 PRBC unit postoperatively. Conversely, sex (male) and preoperative haematocrit generated a negative coefficient, signifying that an increase in preoperative haematocrit is associated with a decrease in the use of PRBC, and males have a lower risk of using PRBC compared to females (Supplementary Material, Table S4). All other variables were not significant in the final model.

**Figure 5:**
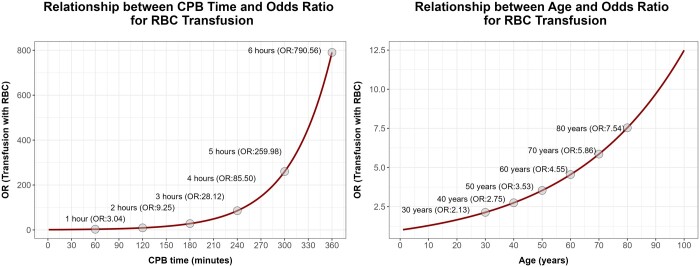
Visual representation of the relation between odds ratio (OR) for the use of transfusions and (**A**) cardiopulmonary bypass time or (**B**) patient age.

The OR for the variable ‘CPB time’ is 1.019 (1.012–1.026, 95% CI). With 1 h of CPB, the OR increases to 3.04, with 2 h to 9.25, then rising exponentially to an OR of 85.5, 259.98 and 790.56 with 4, 5 and 6 h of CPB, respectively (Fig. 5). The same trend is observed for ‘age’, which has an OR of 1.026 (1.006–1.046, 95% CI). For example, if we consider a patient with 40 years, the OR for a single PRBC is 2.75, rising to 4.55 and 7.54 with 60 and 80 years, respectively (Fig. 5). The OR for ‘final ACT’ was 1.021 (1.010–1.032, 95% CI).

We then estimated the predicted probabilities for the model’s dependent variable (PRBC use) by patient sex and surgery timing (Supplementary Material, Table S5), considering the hypothetical scenario in which CPB time, final ACT, preoperative haematocrit and age correspond to the median value of our population (62 min, 121 s, 40.2% and 71 years, respectively).

**Figure 6:**
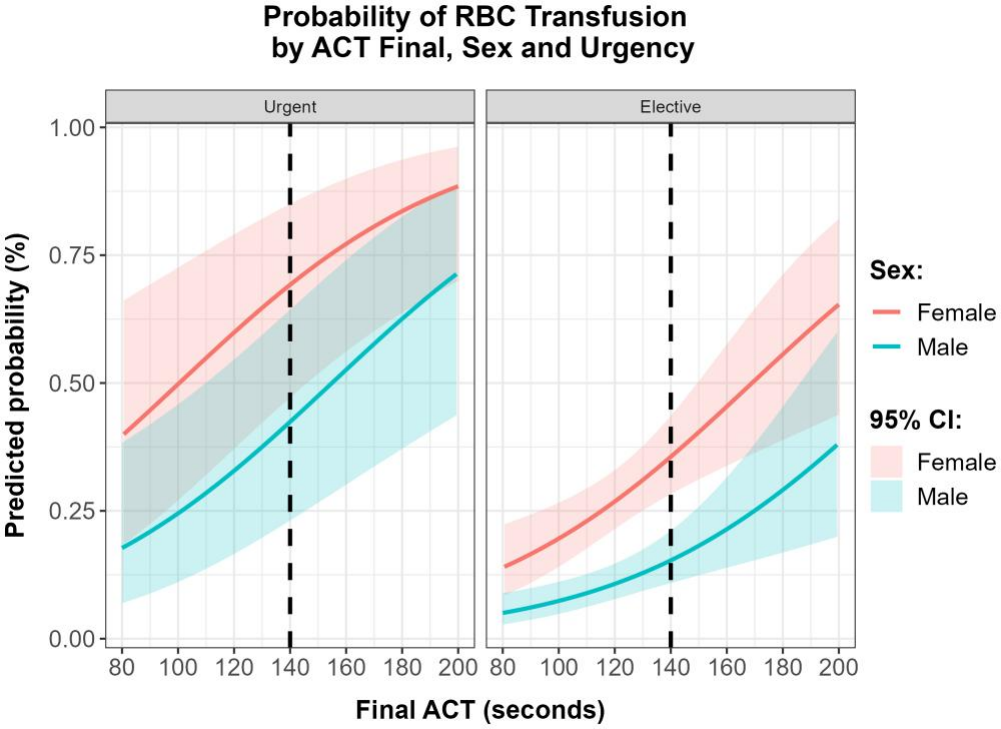
Line charts with 95% confidence interval bands with the relation between the use of transfusions and final activation clotting time (ACT) for males (blue) and females (pink) in the case of urgent (left) and elective (right) surgeries.

The predicted probability for the use of PRBC in females is, according to this model, 27.23% (21.84–33.39%, 95% CI) in elective and 60.38% (37.65–79.36%, 95% CI) in urgent surgeries. In males, the predicted probability for the use of PRBC is 10.92% (7.89–14.92%, 95% CI) in elective and 33.29% (16.89–55.08%, 95% CI) in urgent surgeries. Figure 6 shows the predicted probability of PRBC use by final ACT, sex and surgery timing, assuming median values for the other variables.

A ROC curve was elaborated for the binary logistic regression model used (Supplementary Material, Fig. S4), with an AUC value of 0.854, indicating that the model performed well in distinguishing between the 2 categories assessed (use versus non-use of PRBC).

## DISCUSSION

Despite recent advances in surgical techniques, CPB, anaesthesia management and postoperative care, postoperative bleeding remains a prevalent complication after cardiac surgery. Severe or massive blood loss occurs in approximately 10% of all patients submitted to cardiac surgery [5]. Thus, PRBC transfusion is common after cardiac surgeries, accounting for 10–15% of all PRBC transfusions in the UK and the United States of America [5, 15, 16]. Despite ongoing improvements, there has been only a modest decline in transfusion use over the last decade, with more than 50% of high-risk patients still requiring transfusions [5, 17].

However, although transfusions are essential, they are far from being innocuous. Addressing postoperative bleeding to reduce the use of transfusions can improve surgical outcomes, and may decrease hospital-associated costs [1, 17, 18].

Here, we identified CPB time as a predictor for both significant bleeding and the need for PRBC transfusion. Specifically, the risk of significant bleeding increases 1.009 times with each minute of CPB time, and similarly, the risk of PRBC transfusion rises by 1.019 times with each minute of CPB time. While these values might not appear notably high at first glance, it is crucial to consider that the average time of cardiac ranges from 3 to 5 h (with a considerable part using CPB), exponentially increasing the risk of significant bleeding and use of transfusion.

Another impactful variable in postoperative bleeding and the use of transfusion is preoperative haematocrit, frequently reduced in cardiac surgery patients. Approximately 25–30% of patients undergoing cardiac surgery have multifactorial preoperative anaemia [5, 19]. According to our model, preoperative haematocrit is a predictor of significant bleeding, indicating that patients with higher haematocrit values within physiological range have a lower risk of bleeding, regardless of patient sex. Consistent with our findings, a study involving patients submitted to CABG demonstrated that low preoperative haematocrit levels were associated with increased blood drainage during and after surgery, as well as with increased need for blood transfusion and prolonged intensive care unit stay [20]. These results raise the discussion about whether patients should be transfused preoperatively or have a conservative approach. Exploring this topic, a recent study by LaPar *et al.* [21] involving 33 411 patients from 19 cardiac surgery centres, revealed that PRBC transfusion was a stronger risk factor for morbidity and mortality than preoperative haematocrit level, supporting efforts to minimize unnecessary PRBC transfusions.

Our study also observed that men have a higher risk of significant postoperative bleeding that is on average 2.842 times higher than females, with males having a 15.89% probability of significant bleeding, more than twice the 6.23% probability observed in females. Conversely, females had a higher likelihood of requiring postoperative transfusions compared to males, irrespective of surgery timing. In elective surgeries, the probability of transfusion use for females was 27.23%, nearly 3 times higher than the 10.92% for males. Similarly, in urgent surgeries, females had an almost 2 times higher probability of requiring transfusion compared to males (60.38% vs 33.29%, respectively). These results are supported by previous studies, which demonstrated that regardless of tranexamic acid use, females had less blood loss after CABG, although the risk for postoperative transfusion was higher [22].

A recent meta-analysis, although only including patients undergoing isolated CABG, revealed that females face a higher risk of operative and late mortality compared to males. Additionally, females exhibit a heightened risk of non-fatal occurrences, including major adverse cardiac events, myocardial infarction and stroke [23]. Considering that transfusions are not innocuous, with several studies finding that the transfusion of a single PRBC increases morbidity (higher rate of stroke, respiratory complications, acute kidney injury, among others) and mortality in patients undergoing cardiac surgery [1, 5, 24], the elevated risk of postoperative transfusion in females may contribute, at least partially, to the observed consequences in morbimortality within this sex group. Further studies might explore whether these findings are consistent across different types of cardiac surgery and investigate the biological mechanisms that underlie these differences.

Unsurprisingly, our findings indicate that surgery timing influences the use of transfusion, regardless of patient sex. The probability of transfusion use in urgent surgeries, compared to elective procedures, was >2 times higher in females (60.38% vs 27.23%, respectively) and ∼3 times higher in males (33.29% vs 10.92%, respectively). Several factors may contribute to these disparities, including sex-specific differences in heart physiology and anatomy, as well as variations in the pharmacokinetics and pharmacodynamics of cardiovascular drugs [25–27].

We have emphasized the significance and utility of ACT as a parameter for assessing the degree of anticoagulation, highlighting its advantages in considering various elements in blood that contribute to thrombus formation and its suitability as a point-of-care test, unlike most laboratory test. Despite the use of a recommended value for anticoagulation during CPB, it is based on limited data. There is scant evidence supporting the ideal ACT value after protamine administration, and what the ACT value at the end of cardiac surgeries should be. Currently, the most widely employed strategy involves matching final ACT values to those before heparin administration, although some data suggest that a lower final ACT than the basal ACT might result in less bleeding and fewer postoperative transfusions [12].

In our study, we observed a statistically significant difference in bleeding between patients who had final ACT values ≥140 s and those who had final ACT values <140 s, with the former experiencing a higher bleeding rate in the first 12 h after surgery. Our data suggest that differences in bleeding, possibly related to final ACT, are most evident during the first 12 h after surgery. However, final ACT is not a robust predictor for significant bleeding, regardless of whether it is compared to the patient’s basal ACT or to the fixed reference value of 140 s.

While the final ACT value may not predict the occurrence of significant bleeding, our model revealed that it is a predictor for the need for transfusion, with the risk of transfusion use increasing 1.021 times with each second in the final ACT. Furthermore, patients with a final ACT ≥ 140 s have a 1.81 times higher risk of requiring transfusion compared to those with a final ACT < 140 s.

Considering the substantial impact of bleeding and transfusions on patient morbimortality, we advocate for tight monitoring of final ACT as the standard of care. The evidence suggests that values under 140 s might be safer, leading to less post-surgical bleeding and lower PRBC use. While our study provides valuable insights, we acknowledge the need for additional prospective multicentre studies with larger patient cohorts to determine the ideal final ACT. It is noteworthy that the observed discrepancy between the predictive capacity of final ACT for transfusion versus significant bleeding may be influenced by the chosen cut-off of 140 s; exploring different cut-off values in future studies could yield different conclusions.

### Limitations

This study has several limitations. This is a retrospective, observational, single-centre study, which may limit the generalization of our findings. Observational studies are open to confounders and bias. The results from our study may not be extrapolated to other different populations. The evidence on ACT may exhibit variability depending on the machines used, as different machines employ different methods for clot activation and detection, potentially leading to considerable measurement variability and precluding the interchangeability of values from different machines. The sample size is another potential limitation.

## CONCLUSION

Concluding, patients with final ACT ≥140 s exhibit significantly higher postoperative bleeding, particularly notable in the first 12 h after surgery, and a heightened risk of transfusion compared to those with final ACT value <140 s. Final ACT has a good predictive value for the use of transfusions, although it is not a robust predictor for significant bleeding. The risk of bleeding and transfusion use is amplified with prolonged time under CPB and lower preoperative haematocrit values. Males face a higher postoperative risk of bleeding, while females have a greater risk of transfusion use. Surgery timing influences transfusion use, with patients undergoing urgent surgery at a higher risk for PRBC use than those with elective procedures.

## Supplementary Material

ivae092_Supplementary_Data

## Data Availability

The derived data generated in this research will be shared on reasonable request to the corresponding author.

## ABBREVIATIONS

ACT: Activated clotting time
AUC: Area under the curve
CABG: Coronary artery bypass graft
CI: Confidence interval
CPB: Cardiopulmonary bypass
OR: Odds ratio
ROC: Receiver operating characteristic
