# Supplementary material for: Activated clotting time value as an independent predictor of postoperative bleeding and transfusion
Source: Interdiscip Cardiovasc Thorac Surg. 2024 May 8;38(5):ivae092. doi: 10.1093/icvts/ivae092 (PMC11109492; doi:10.1093/icvts/ivae092)
Supplement: ivae092_Supplementary_Data [file ivae092_supplementary_data.zip › Supplementary Tables.docx]

**Supplementary Table 1 – Estimated effects of the independent variables Time and Group on bleeding volume using a linear mixed regression analysis**

|  | **Estimate** | **Std. Error** | **df** | **t value** | **Pr(>⏐t⏐)** |  |
| --- | --- | --- | --- | --- | --- | --- |
| **(Intercept)** | 1.828 | 13.865 | 1559.680 | 0.132 | 0.89512 |  |
| **Time 12h post-op** | 379.384 | 14.187 | 1510.837 | 26.742 | <0.001 | *** |
| **Time 24h post-op** | 560.088 | 14.188 | 1510.208 | 39.476 | <0.001 | *** |
| **Group≥140s:Time12h post-op** | 99.616 | 36.386 | 1510.125 | 2.738 | 0.00626 | ** |
| **Group≥140s:Time24h post-op** | 104.999 | 36.387 | 1510.029 | 2.886 | 0.00396 | ** |

The calculated R-square was 0.65 (65%), from which 0.32 came from fixed effects and 0.32 came from random effects.The RMSE (Root Mean Squared Error) and the MAE (Mean Absolute Error) were used to assess the quality of data adjustment. The present model was the one in which the lowest values of RMSE (220.63 ml) and MAE (133.78 ml) were presented, indicating that it was the best model adjustment to the data. The analysis resulting from the comparison of the residual values’ dispersion versus the adjusted values’ allowed to verify that there were no trend patterns or non-linearities that could have meant problems in model adequacy. In general, the residuals are randomly scattered around zero, with no clear trend that could indicate a problem in model specification or variable selection. df: degrees of freedom; Post-op: postoperative; Std: standard.

**Supplementary Table 2 – Estimated postoperative bleeding volume at 12h and 24h for patients with Final ACT ≥ 140 seconds and Final ACT < 140 seconds**

| **Group** | **Predicted (mL)** | **Std. Error** | **Conf. Low** | **Conf. High** |
| --- | --- | --- | --- | --- |
| **12h post-op <140s** | 379.38 | 14.19 | 354.04 | 408.38 |
| **12h post-op ≥140s** | 479.00 | 36.39 | 415.79 | 544.21 |
| **24h post-op <140s** | 560.09 | 14.19 | 534.74 | 589.09 |
| **24h post-op ≥140s** | 665.09 | 36.39 | 601.88 | 730.30 |

**Supplementary Table 3 – Coefficients and Odds Ratio of the statistically significant explanatory variables, using a binary logistic regression to explore the relation between significant bleeding and possible relevant variables**

|  | **Estimate** | **Std. Error** | **Z value** | **Pr(>⏐t⏐)** |  | **OR** | **95% CI** |
| --- | --- | --- | --- | --- | --- | --- | --- |
| **(Intercept)** | -0.213 | 0.975 | -0.218 | 0.82707 | *ns* | 0.808 | 0.117 – 5.399 |
| **CBP Time** | 0.009 | 0.003 | 2.772 | 0.00557 | ** | 1.009 | 1.002 – 1.015 |
| **Preoperative Hematocrit** | -0.076 | 0.025 | -2.964 | 0.00303 | ** | 0.927 | 0.882 – 0.975 |
| **Sex (male)** | 1.044 | 0.262 | 3.990 | <0.001 | *** | 2.842 | 1.721 – 4.821 |

CI: confidence interval; CPB: cardiopulmonary bypass; OR: odds ratio; Pr: probability

**Supplementary Table 4 – Coefficients and Odds Ratio of the statistically significant explanatory variables, using a logistic regression to explore the relation between the use of at least one transfusion and possible relevant variables**

|  | **Estimate** | **Std. Error** | **Z value** | **Pr(>⏐t⏐)** |  | **OR** | **95% CI** |
| --- | --- | --- | --- | --- | --- | --- | --- |
| **(Intercept)** | 4.012 | 1.684 | 2.382 | 0.01722 | *** | 55.230 | 2.075 – 1546.331 |
| **CBP Time** | 0.019 | 0.003 | 5.436 | <0.001 | *** | 1.019 | 1.012 – 1.026 |
| **Final ACT** | 0.021 | 0.006 | 3.590 | <0.001 | *** | 1.021 | 1.010 – 1.032 |
| **Preoperative Hematocrit** | -0.290 | 0.030 | -9.645 | <0.001 | *** | 0.748 | 0.704 – 0.792 |
| **Urgent (Non-elective)** | 1.382 | 0.472 | 2.927 | 0.00342 | ** | 3.982 | 1.589 – 10.195 |
| **Age** | 0.025 | 0.010 | 2.573 | 0.01008 | * | 1.026 | 1.006 – 1.046 |
| **Sex (male)** | -1.088 | 0.220 | -4.950 | <0.001 | *** | 0.337 | 0.218 – 0.516 |

ACT: activated clotting time; CI: confidence interval; CPB: cardiopulmonary bypass; OR: odds ratio; Pr: probability

**Supplementary Table 5 – Predicted probabilities and their respective 95% CI for transfusions use based on patient’s sex and surgery timing**

| CPB Time | Final ACT | Pre Htc | Age | Urgency | Sex | PP | 95% CI |
| --- | --- | --- | --- | --- | --- | --- | --- |
| 62 | 121 | 40.2 | 71 | Elective | Male | 10.92 | 7.89 – 14.92 |
| 62 | 121 | 40.2 | 71 | Elective | Female | 27.23 | 21.84 – 33.39 |
| 62 | 121 | 40.2 | 71 | Urgent | Male | 33.29 | 16.89 – 55.08 |
| 62 | 121 | 40.2 | 71 | Urgent | Female | 60.38 | 37.65 – 79.36 |

ACT: activated clotting time; CI: confidence interval; CPB: cardiopulmonary bypass; OR: odds ratio; Pre Htc: preoperative hematocrit; PP: predicted probability
