# Supplementary material for: Activated clotting time value as an independent predictor of postoperative bleeding and transfusion
Source: Interdiscip Cardiovasc Thorac Surg. 2024 May 8;38(5):ivae092. doi: 10.1093/icvts/ivae092 (PMC11109492; doi:10.1093/icvts/ivae092)
Supplement: ivae092_Supplementary_Data [file ivae092_supplementary_data.zip › Supplementary Figures.docx]

Supplementary Figure 1


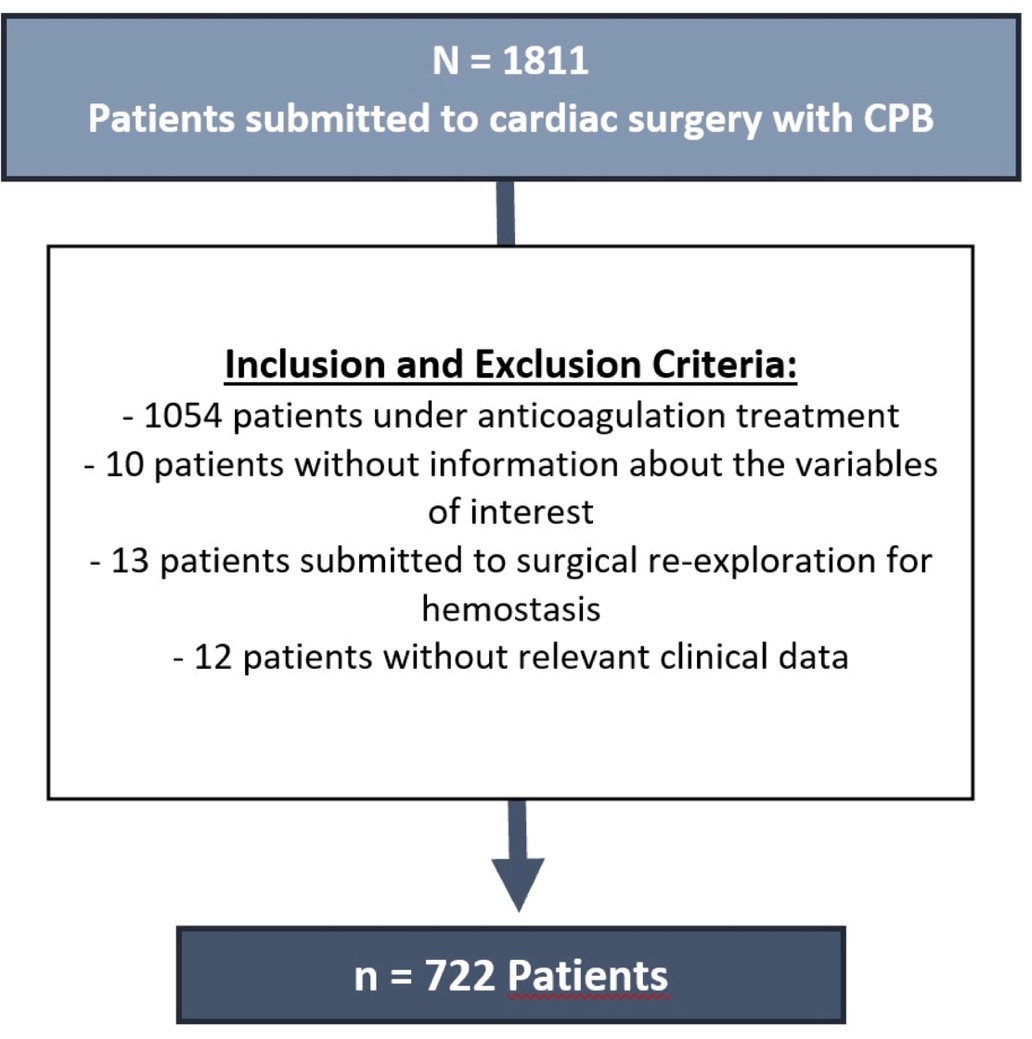


Supplementary Figure 2


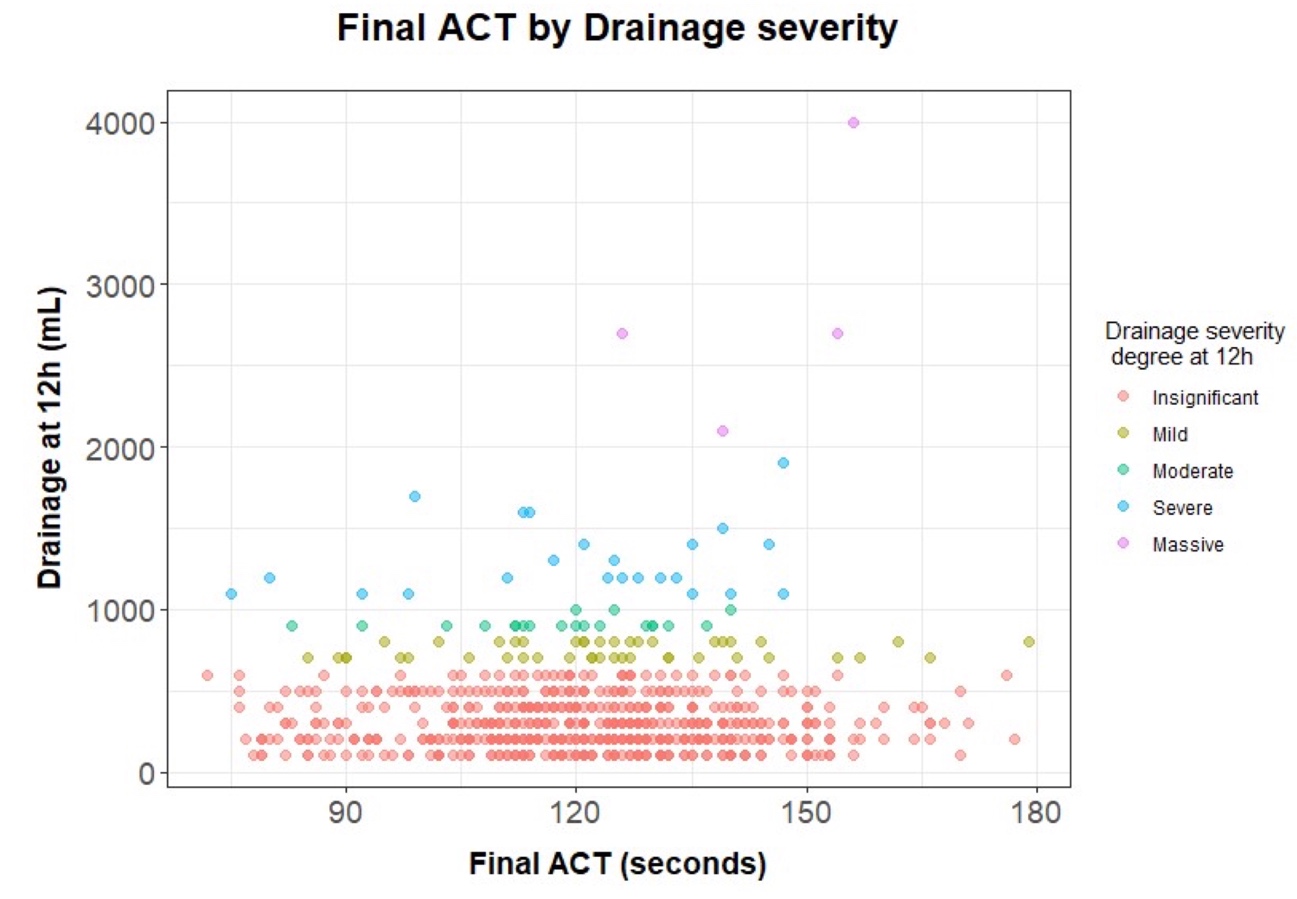


Supplementary Figure 3


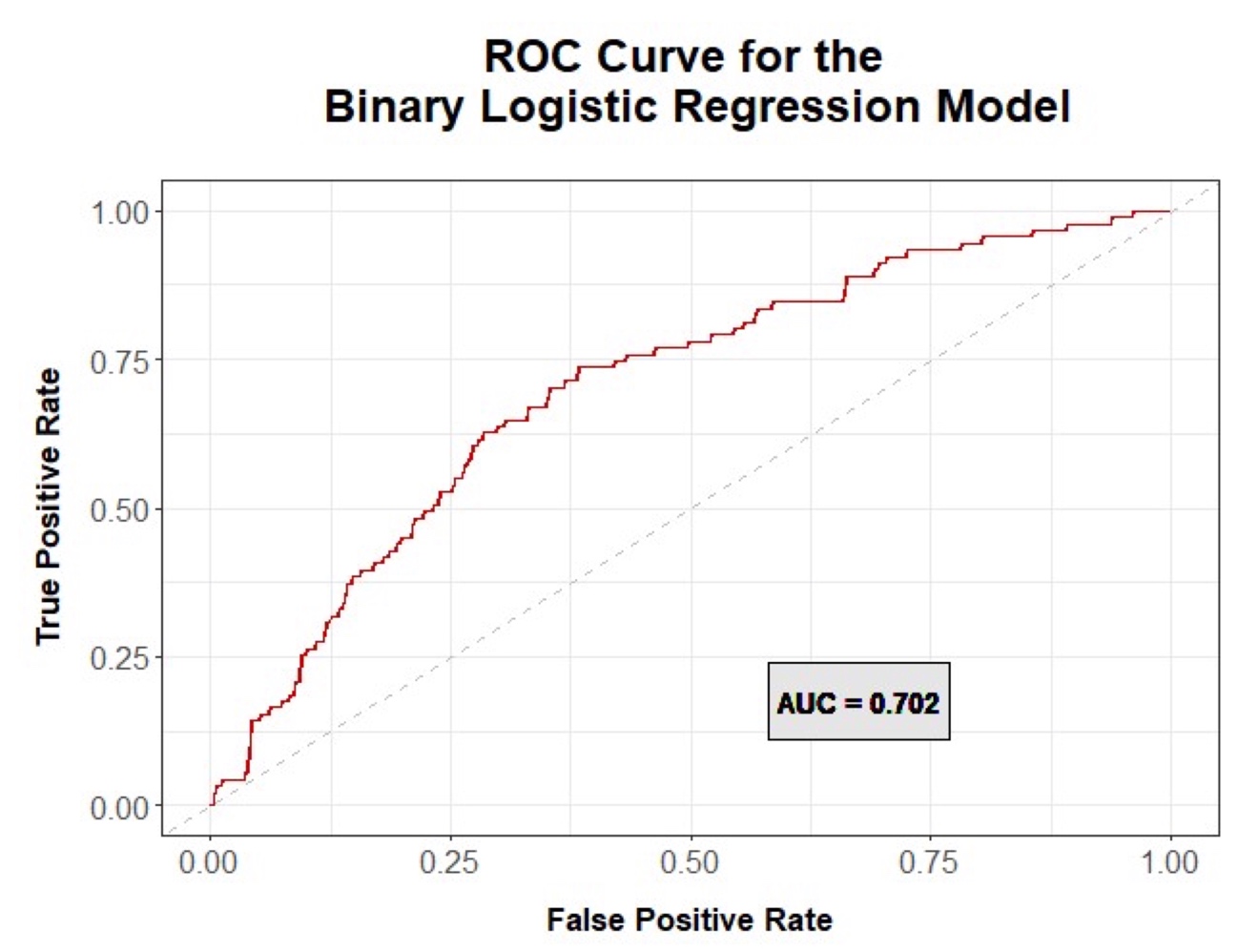


The assumptions for the binary logistic regression were verified, including the absence of multicollinearity of the explanatory variables. The observations that stood out were analyzed and the existence of potentially influential and effectively influential observations was assessed using the Cook distance, with which we verified the absence of distances greater than 0.5. In addition, the Hosmer & Lemeshow test was performed to evaluate model suitability, having found the model adequate (p = 0.9910).

Supplementary Figure 4


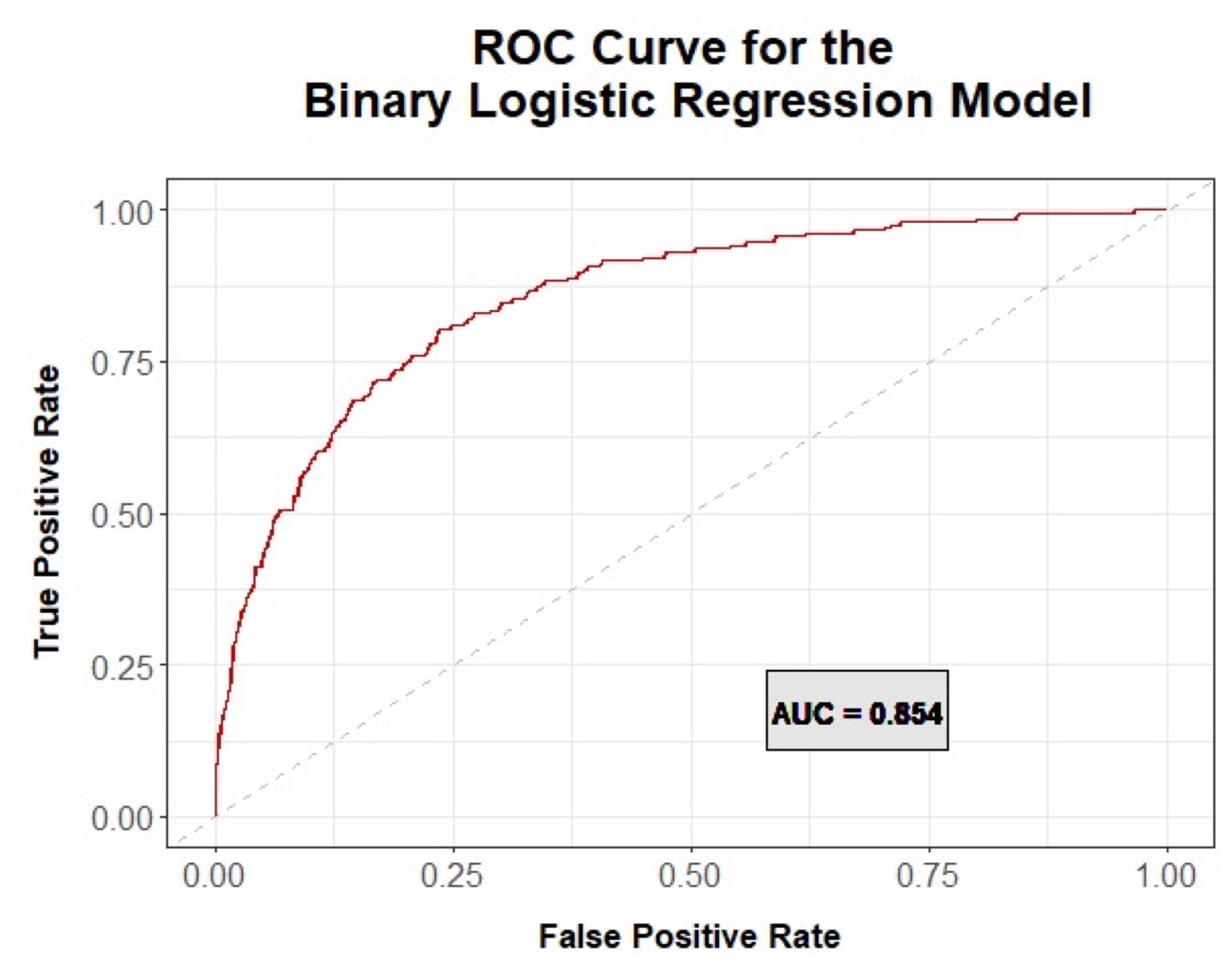


The assumptions for the binary logistic regression were verified, including the absence of multicollinearity of the explanatory variables. The observations that stood out were analyzed and the existence of potentially influential and effectively influential observations were evaluated using the Cook distance, with which we verified the absence of distances greater than 0.5. In addition, the Hosmer & Lemeshow test was performed to evaluate model suitability, and it was found that the model is adequate (p = 0.9999).
